# The Use of Magnetic Resonance Imaging in Patients With Shoulder Pain at King Abdulaziz University Hospital

**DOI:** 10.7759/cureus.77983

**Published:** 2025-01-25

**Authors:** Ahmed Abduljabbar, Heba E Mahboob, Taif H Mubarak, Jumanah M Baalawi, Ghadah A Albashrawi, Rawan N Alharbi

**Affiliations:** 1 Faculty of Medicine, King Abdulaziz University, Jeddah, SAU

**Keywords:** anterior labrum periosteal sleeve avulsion, magnetic resonance imaging, shoulder imaging, shoulder joint, shoulder pain, subacromial subdeltoid bursitis, subscapularis partial thickness tears, supraspinatus tendinopathy

## Abstract

Objective: Our study aims to assess the clinical effectiveness of using MRI in diagnosing various shoulder pain-related conditions among patients at King Abdulaziz University Hospital.

Methods: 383 patients who were admitted to King Abdulaziz University Hospital and had shoulder magnetic resonance imaging between January 2020 and July 2024 were studied retrospectively. The dataset was subjected to a thorough statistical analysis using descriptive and inferential approaches. The participants' demographic details, such as age, gender, and other attributes, were first compiled by a descriptive analysis, and then, a summary of the study population was given.

Results: 383 patients were enrolled in our study for MRI evaluation. Of them, 160 (41.8%) were men and 223 (58.2%) were women. The age ranged from 7 to 91 years, and the mean age was 48.4 years. While 120 (31.3%) individuals reported other problems such as motion limitation, trauma, edema, shoulder instability, or other clinical issues, 262 participants (68.4%) indicated shoulder pain as their primary complaint. 174 participants (45.4%) had their left shoulder MRI, and 209 participants (54.6%) had their right shoulder MRI. In contrast to normal MRI findings (n=24, 6.3%). Abnormal findings were more common (n=358, 93.5%).

Conclusion: Our research further supports the importance of MRI in diagnosing shoulder discomfort and associated disorders. The high frequency of anomalies found by MRI highlights its usefulness as a diagnostic technique. By comparing our results with previous research, we emphasize how crucial it is to combine MRI results with clinical evaluation to inform efficient treatment plans for patients with shoulder disorders. Future studies should focus on resolving existing issues and investigating how sophisticated imaging methods might improve diagnostic precision.

## Introduction

Magnetic resonance imaging (MRI) is an essential diagnostic tool because of its multi-planar imaging, high resolution, high sensitivity in representing the soft tissues, and absence of ionizing radiation. MRI uses the body's natural magnetic properties to produce detailed images of any body part [1,2]. In the general population, shoulder pain is a common cause of morbidity. Differential diagnosis could be challenging. Soft tissue shoulder disorders are the most common causes of shoulder pain. Thus, MRI is considered one of the best diagnostic tools for shoulder pain [3].

Shoulder pain is the third most prevalent musculoskeletal complaint for which people seek medical attention, behind low back pain and knee pain, according to self-reported prevalence in the general population [4]. A thorough systematic review that incorporated data from 61 research conducted in high, middle, and low-income countries looked at the prevalence and incidence of shoulder pain worldwide. The community prevalence of shoulder pain varied considerably across the included countries, with a median of 16% [5]. In Saudi Arabia, shoulder pain due to different pathologies has been prominent in several areas. Among 378 participants, a study conducted in Taif City found that the prevalence of shoulder pain is 32.8% (124) [6].

In a retrospective study conducted by Reynolds et al. (2017) of all new patient visits for shoulder pain to an academic medical center shoulder and elbow surgery practice between July 2012 and June 2013, 491 patients were included. Of these, 40% had an MRI study before presenting to the shoulder and elbow clinic. The clinic ordered an additional 79 MRI studies for patient evaluation and treatment. For all 275 patients evaluated with MRI, 36% did not receive any treatment with injections or physical therapy before imaging, and 66% did not have any surgery. The results of this study highlight the opportunity for increased discretion to be used when ordering MRI studies for patients presenting with shoulder pain. Although each case requires unique clinical judgment, general trends can help identify patient groups where such discretion could be particularly warranted. One in four patients who had an MRI ordered to evaluate their shoulder pain did not have any formal treatment beforehand and also did not receive surgical treatment. This category of patients was mainly those diagnosed with partial thickness rotator cuff tear, rotator cuff tendinitis, benign shoulder pain, or arthritis. For this subgroup of patients, a well-focused physical exam, steroid injection, or trial of physical therapy could significantly reduce the number of patients requiring an MRI to be successfully treated [7].

In Nairobi, Onyambu et al. (2014) conducted a cross-sectional investigation at three imaging centers to determine the pattern of results found on MRI in patients with shoulder discomfort. The three imaging centers scanned 70 individuals referred for MRI. The scan date, age, gender, and referring doctor were all recorded. The majority of individuals submitted for evaluation had pathology in their right shoulder. Tendinosis was the most prevalent lesion, accounting for 47.2% of cases, and was more common on the right shoulder. There were 27.8% rotator cuff tears that primarily involved the supraspinatus tendon. Degenerative disease was found in 18.6% of patients [8].

MRI is a trustworthy technology for determining the various reasons for shoulder discomfort. The architecture of the shoulder is unusual, having remarkable flexibility and range of motion. There are several reasons for painful shoulder syndrome, the most common of which is shoulder impingement, which various conditions can cause. Allam et al. (2019) sought to review a reliable and highly sensitive diagnostic examination of often observed acute shoulder joint injuries in 40 athletes experiencing shoulder pain. The most frequent MRI findings were effusion, Bankart, Hillsachs, and rotator cuff tears. The majority of cases had two to three findings. There is no significant difference according to the mode of trauma regarding age and sex. Weight lifting was significantly the most frequent regarding traction mode of trauma; team sport was significantly the most frequent regarding direct impact mode of trauma, while weight lifting and team sport had non-significant differences regarding falls on outstretched hands (FOOSH) [9].

In a study by Lazik-Palm et al. (2020), eight patients with suspected rotator cuff lesions underwent 7-T MRI before arthroscopy to evaluate the feasibility and diagnostic performance of clinical 7-T MRI. Regarding image quality, most sequences reached values above the middle of each scoring scale. Fat-saturated proton density sequences produced the fewest artifacts and had the highest structural assessability. Gradient-echo sequences produced the most homogeneous B1+ field. Arthroscopy failed to establish tendinopathy/partial tear of the supraspinatus in 5/8 individuals, the subscapularis in 5/6, and the infraspinatus in one patient; only a partial lesion of the subscapularis tendon was overlooked. Pathologic outcomes of the long bicipital tendon, glenohumeral cartilage, acromioclavicular joint, labrum, and subacromial subdeltoideal bursa were mostly confirmed; one long bicipital tendon lesion, one superior glenoid labrum anterior-to-posterior lesion, and one subacromial bursitis were not detected on 7-T MRI. When all structures were evaluated collectively, the sensitivity was 86%, and the specificity was 74%. There was greater contrast and picture resolution compared to prior 1.5-T tests. The research demonstrated a 7-T MRI of the shoulder with diagnostic picture quality. The overrating of tendon signal alterations was the main limitation [10].

Shoulder pain is a common social complaint related to various illnesses, and determining the best diagnostic imaging modality to identify the actual cause is an important step in treatment and prognosis. This research aims to determine the percentage of MRI sensitivity in diagnosing shoulder pain-related illnesses, the reliability of using noninvasive techniques in diagnosing shoulder tenderness over invasive tests, and the accuracy of localizing the injury at the level of the shoulder joint in specific populations that visit the King Abdulaziz University Hospital's (KAUH) radiological department for diagnosis and treatment of shoulder pain.

## Materials and methods

Patients and study design

This retrospective record review was conducted at King Abdulaziz University (KAUH), a tertiary center in Jeddah, Saudi Arabia, from January 2020 to July 2024. The medical records of 408 patients who had magnetic resonance imaging (MRI) at KAUH between 2020 and 2024 were obtained. Of these, 383 patients' records were studied. From the original sample, 25 patients were excluded because they had either missing data, a scheduled MRI was not performed due to claustrophobia or another reason, or an MRI was performed as a follow-up after surgery. 

Data collection

Data was gathered electronically from patients' hospital files and radiology reports. Data was collected, and each patient's computerized form was filled out separately. The data collecting form contained the patient's personal information, shoulder side (left or right), a specific clinical question (indication for the MRI), and findings of the MRI.

Outcome

The study's primary outcome was calculating the prevalence of pathologies identified by MRI in a clinical question. 

Statistical analysis

A comprehensive statistical analysis was conducted on the dataset, encompassing descriptive and inferential methodologies. Firstly, a descriptive analysis is conducted to summarize the participants' demographic characteristics, including age, gender, shoulder side, presenting complaints, and MRI findings. This provides an overview of the study population. Subsequently, the Chi-Square and Fisher's Exact Test are used to see the association between categorical variables. Statistical significance is established at a p-value of 0.05 or lower and a 95% Confidence Interval. All statistical analyses are executed using IBM's SPSS Software, version 29.0.0. 

Ethical considerations

An institutional board review approval was obtained from King Abdulaziz University's Unit of Biological Ethics (Approval Reference Number: 455-24, NCBE Number: HA-02-J-008). Due to the retrospective nature of the study, informed consent was not required. 

## Results

**Figure 1:**
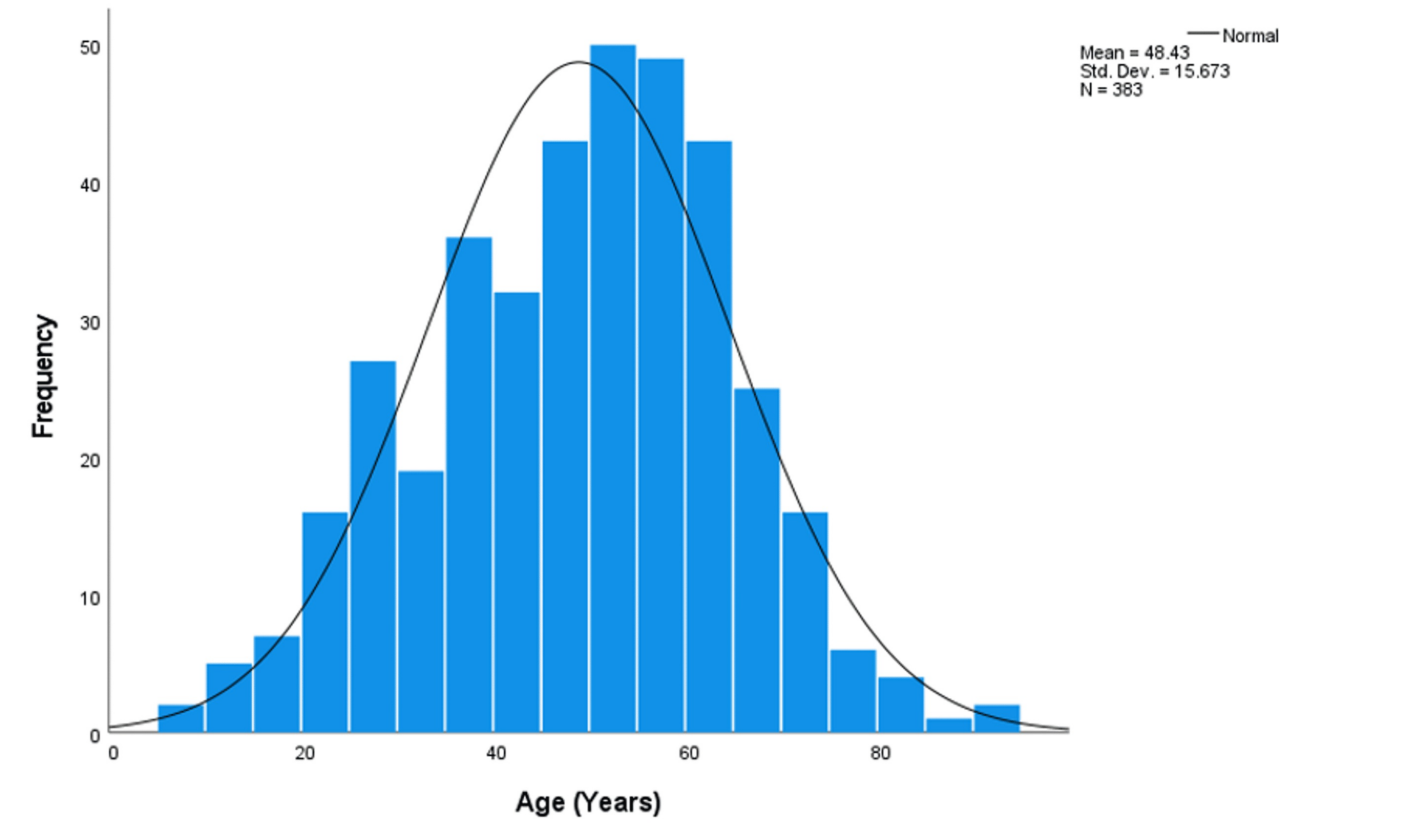
Distribution curve of age of participants.

Our study included 383 participants for MRI assessment. Among them, 223 (58.2%) were female, and 160 (41.8%) were male. The mean age was 48.4 years (SD 15.6) (Figure 1 shows the distribution of Age), ranging from 7 to 91 years, with the majority aged 41-60 years (n=180, 47.0%)

Shoulder pain was the primary complaint for 262 participants (68.4%), while 120 (31.3%) reported other complaints such as motion limitation, trauma, swelling, shoulder instability, or other clinical questions. MRI scans were conducted on the left shoulder for 174 participants (45.4%) and on the right shoulder for 209 participants (54.6%). Abnormal MRI findings were prevalent (n=358, 93.5%) compared to normal findings (n=25, 6.5%) (Table 1). 

**Table 1:** Sociodemographic and other parameters of participants.

Variables		Frequency (n=383)	Percentage
Gender	Female	223	58.2
	Male	160	41.8
Age (Years)	<20 Years	14	3.7
	21-40 Years	103	26.9
	41-60 Years	180	47.0
	61-80 Years	80	20.9
	>80 Years	6	1.6
	Mean (SD)	48.4 (15.6)	
	Range	7-91	
Presenting complaints	Pain of Shoulder	262	68.4
	Other Complaints	120	31.3
MRI of shoulder	Left	174	45.4
	Right	209	54.6
MRI findings	Normal	25	6.35
	Abnormal	358	93.5

**Figure 2:**
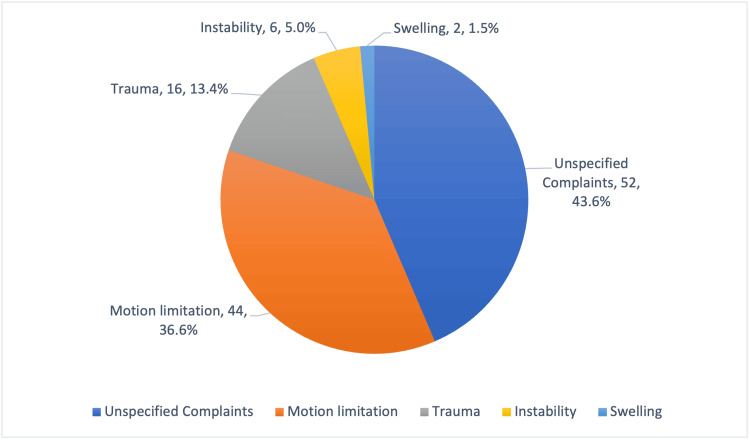
Different other types of presenting complains of participants (n=120).

Figure 2 shows the distribution of various other types of presenting complaints among participants. The most common unspecified complaints were reported by 52 participants (43.6%). Motion limitation was the second most frequent complaint, accounting for 44 participants (36.6%). Trauma-related complaints were noted in 16 participants (13.4%), while instability was reported by 6 participants (5.0%). Swelling was the least common complaint, with 2 participants (1.5%) reporting it.

**Figure 3:**
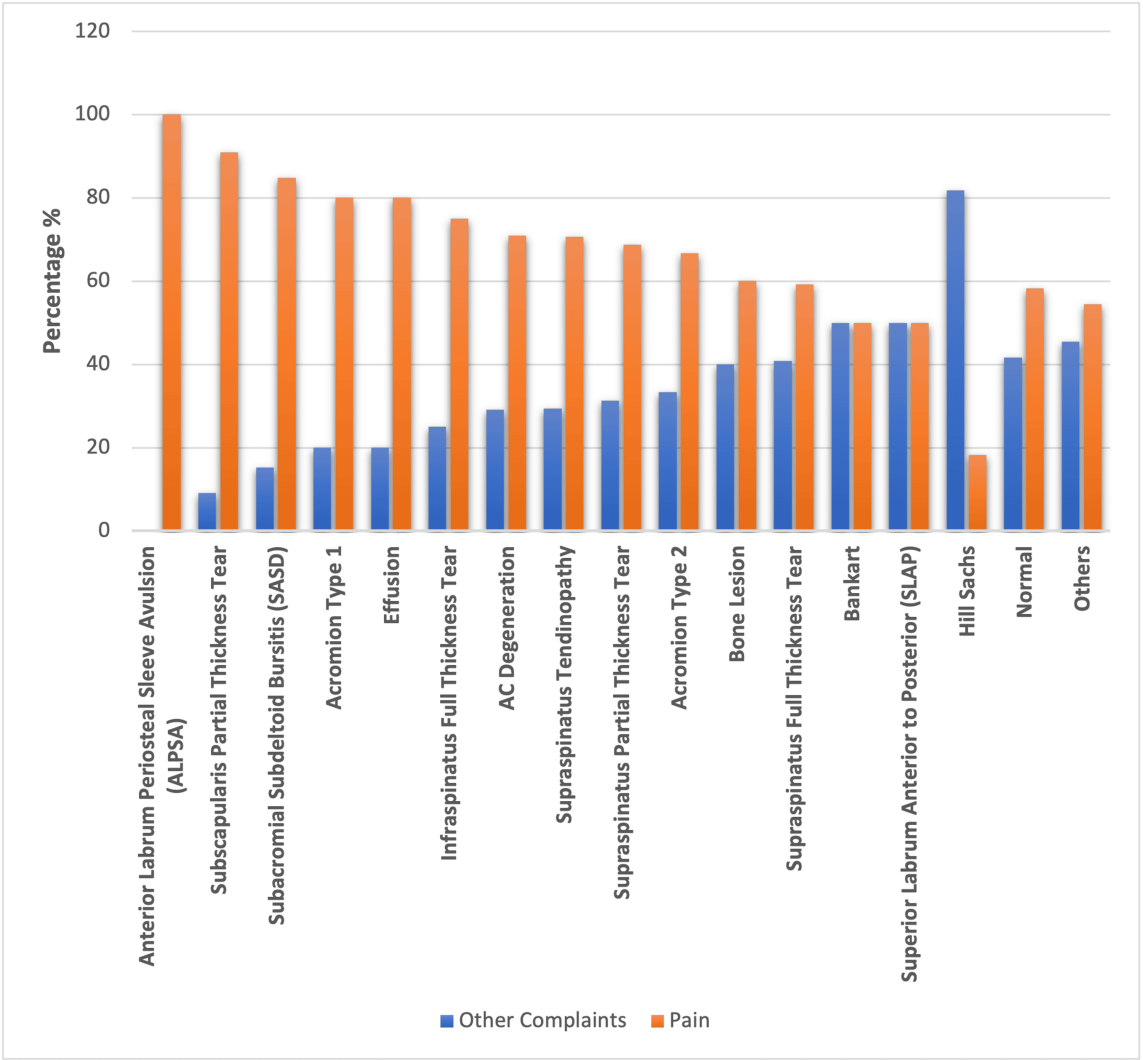
Different MRI findings of patients with shoulder pain and other complains of shoulder.

Figure 3 shows the distribution of various MRI findings among patients with shoulder pain and other shoulder complaints. The findings are listed as; anterior labrum periosteal sleeve avulsion (ALPSA): was observed in 2 patients, and all of them presented with shoulder pain (100%); subscapularis partial thickness tear: 90.9% of those who have subscapularis partial thickness tear presented with shoulder pain; subacromial subdeltoid bursitis (SASD): 84.8% of patients with SASD presented with shoulder pain; acromion type 1: 16 (80%) patients of acromion type 1 complained of shoulder pain; effusion: 16, (80%) effusion patients, complained of shoulder pain; supraspinatus tendinopathy: supraspinatus tendinopathy with shoulder pain complaints was noted in 12 (70.6%) patients ; AC degeneration: 39, (70.9%) AC degeneration patients, presented with shoulder pain symptoms; supraspinatus partial thickness tear: shoulder pain was observed in 55 (68.8%) patients; acromion type 2: 12 (66.7%) patients reported of shoulder pain; bone lesion: 3 (60%) patients with bone lesions were identified having shoulder pain; supraspinatus full thickness tear: 29 (59.2%) patients of supraspinatus full thickness tear were diagnosed having shoulder pain as a presenting symptom; Bankart lesion: 2, (50%) patients with Bankart lesions presented with shoulder pain; superior labrum anterior to posterior (SLAP): 3 (50%) patients with SLAP had shoulder pain; Hillsachs Lesion: was the least common abnormality with only two shoulder pain patients (Figure 3).

These findings, as presented in the above figure, emphasize that ALPSA, SASD, and subscapularis partial thickness tear are most frequently associated with shoulder pain.

Table 2 shows the association between patients’ complaints of shoulder pain and various features. Shoulder pain is more frequently associated with abnormal MRI findings, where 66.3% (n=116) of left shoulder MRIs and 70.2% (n=146) of right shoulder MRIs belong to patients reporting shoulder pain; however, the association is not statistically significant (p=0.462). MRI findings also indicate no significant correlation with shoulder pain (p=0.259). Among patients with shoulder pain, 56% (n=14) have normal findings, while 69.3% (n=248) exhibit abnormal findings. Similarly, those with other complaints show abnormal findings in 30.7% (n=110). Overall, the analysis reveals no statistically significant association between shoulder pain and MRI laterality or findings.

**Table 2:** Association between patients’ complaint of pain and different features. (a) Chi-Square test; * Calculated using Cramer's V formula.

			Major complaints of patients		Degrees of freedom	* Effect size	Significance value
			Others	Shoulder Pain			
MRI shoulder	Left	N	59	116	1	0.040	0.462 ^a^
		%	33.7%	66.3%			
	Right	N	62	146			
		%	29.8%	70.2%			
MRI findings	Normal	N	11	14	1	0.062	0.259^ a^
		%	44%	56%			
	Abnormal	N	110	248			
		%	30.7%	69.3%			

Table 3 shows the association between MRI findings in patients with shoulder pain and various demographic and clinical features. Age significantly correlates with abnormal MRI findings (p=0.007). Abnormal findings are more prevalent in older age groups, with 96.9% (n=124) of patients aged 41-60 years, 100.0% (n=51) of those aged 61-80 years, and 100.0% (n=4) of those over 80 years showing abnormalities, compared to younger groups where 77.8% (n=7) of patients under 20 years exhibit abnormalities. Gender does not show a significant association (p=0.396), with abnormal findings observed in 95.7% (n=157) of females and 92.9% (n=91) of males. Shoulder laterality also shows no significant relationship (p=0.167), with abnormal findings on the left in 92.2% (n=107) of patients and on the right in 96.6% (n=141). Age remains the strongest determinant of abnormal MRI findings.

**Table 3:** Association between MRI findings of patients with complaint of pain and other features. (b) Fisher’s Exact test.

			MRI findings		Significance value
			Normal	Abnormal	
Age	<20 Years	N	2	7	0.007^ b^
		%	22.2%	77.8%	
	21-40 Years	N	8	62	
		%	11.4%	88.6%	
	41-60 Years	N	4	124	
		%	3.1%	96.9%	
	61-80 Years	N	0	51	
		%	0.0%	100.0%	
	>80 Years	N	0	4	
		%	0.0%	100.0%	
Gender	Female	N	7	157	0.396^ b^
		%	4.3%	95.7%	
	Male	N	7	91	
		%	7.1%	92.9%	
Shoulder MRI	Left	N	9	107	0.167^b^
		%	7.8%	92.2%	
	Right	N	5	141	
		%	3.4%	96.6%	

## Discussion

Magnetic resonance imaging (MRI) is a key diagnostic tool for shoulder pain due to its high resolution and absence of ionizing radiation [11]. According to Florkow et al. (2022), it is superior to visualize soft tissue structures compared to other imaging modalities [12]. Studies reveal shoulder pain is a prevalent complaint, with MRI providing sensitivity in diagnosis [13]. It can detect common issues like rotator cuff tears and shoulder impingement [14]. Various studies emphasize careful MRI use to avoid unnecessary procedures, highlighting MRI's effectiveness in accurately diagnosing shoulder conditions compared to traditional methods [15]. This study aimed to evaluate the use of MRI at King Abdulaziz University Hospital (KAUH) in diagnosing shoulder pain and related conditions. Our findings highlight critical insights into patients' demographic and clinical profiles and the prevalence of MRI-detected abnormalities.

Notably, our study shows a higher prevalence of female participants (58.2%) compared to males (41.8%). Similarly, Hodgetts et al. (2023) show that females (14.7%) reported shoulder pain in either shoulder more frequently than males (7.7%) [16]. The mean age was 48.4 years, with the majority of patients aged between 41 and 60 years. This demographic distribution aligns with preceding research suggesting that shoulder pain is commonplace in centers of age and older adults, likely because of aging-related degenerative changes. Similarly, a study by Burner et al. (2014) highlights that shoulder pain and dysfunction are common problems in an older adult [17].

Shoulder pain was the primary complaint in 68.4% of participants, with other complaints, including motion limitation, trauma, instability, and swelling, accounting for the rest. MRI findings revealed an overwhelming prevalence of abnormalities (93.5%), significantly higher than normal findings (6.5%). These findings underscore the sensitivity of MRI in detecting shoulder pathologies, corroborating earlier research that emphasizes MRI's superior diagnostic capability over clinical examination alone. Moreover, a study by Thiagarajan et al. (2021) shows that clinical assessment appears to be an effective tool in diagnosing shoulder pathologies, whereas MRI, though reliable in the identification of rotator cuff tears and instability, does not identify patients with SLAP (superior labral tear from anterior to posterior) lesions effectively [18].

Moreover, the most common MRI findings included anterior labrum periosteal sleeve avulsion (ALPSA), subscapularis partial thickness tears, subacromial subdeltoid bursitis, and supraspinatus tendinopathy. ALPSA was identified in all cases of shoulder complaints, indicating its critical role in shoulder instability. Similarly, a case report by Izquierdo et al. (2017) shows that ALPSA is the usual cause of shoulder instability, and MRI is essential for its diagnosis [19]. Notably, certain conditions, such as Hill-Sachs lesions, were more prevalent among patients presenting with shoulder pain, consistent with the traumatic nature of this lesion. Similarly, Alkaduhimi et al. (2021) show that a Hill-Sachs lesion can be detected on radiographic imaging, but computed tomography (CT) and magnetic resonance imaging (MRI) are more sensitive [20].

Notably, age emerged as a significant factor influencing MRI findings, with older age groups demonstrating a higher prevalence of abnormalities. This trend highlights the role of degenerative changes in shoulder pathologies and aligns with studies linking age-related degeneration, which causes higher abnormalities detection on MRI with age [21]. However, gender and the side of the shoulder scanned did not significantly affect MRI outcomes, suggesting that these factors are less influential in the presence of shoulder pathologies.

Implications for clinical practice

The high prevalence of abnormal MRI findings in this study supports the utilization of MRI as a first-line imaging modality in patients with unexplained shoulder pain. The ability of MRI to detect subtle changes in soft tissue structures makes it invaluable in planning surgical interventions and guiding rehabilitation protocols. The study emphasizes the need for clinicians to integrate MRI results with clinical evaluations to enhance diagnostic accuracy and guide effective treatment strategies, ultimately improving patient outcomes. This insight is crucial for avoiding unnecessary delays in diagnosis and treatment, ultimately improving patient outcomes.

Limitations and future directions

While this study provides valuable insights into the use and importance of MRI in shoulder pain, several limitations warrant consideration. The retrospective design may introduce selection bias, and the study's single-center nature limits the generalizability of the findings. Future research should focus on prospective, multi-center studies to validate these findings across diverse populations. Additionally, the role of advanced MRI techniques, such as diffusion-weighted imaging and MR arthrography, in enhancing diagnostic accuracy should be explored. These modalities offer the potential to detect subtle pathological changes that conventional MRI may miss, thereby refining diagnostic precision.

## Conclusions

Our study reinforces the critical role of MRI in diagnosing shoulder pain and related conditions. The high prevalence of abnormalities detected by MRI underscores its value as a diagnostic tool. By aligning our findings with existing literature, we highlight the importance of integrating MRI findings with clinical assessment to guide effective management strategies for patients with shoulder pathologies. Future research should aim to address current limitations and explore the potential of advanced imaging techniques in enhancing diagnostic accuracy.
